# Computational prediction and experimental validation of novel Hedgehog-responsive enhancers linked to genes of the Hedgehog pathway

**DOI:** 10.1186/s12861-016-0106-0

**Published:** 2016-02-24

**Authors:** Katherine Gurdziel, Kyle R. Vogt, Gary Schneider, Neil Richards, Deborah L. Gumucio

**Affiliations:** Department of Cell and Developmental Biology, University of Michigan, Ann Arbor, MI 48109 USA; Department of Computational Medicine and Bioinformatics, University of Michigan, Ann Arbor, MI 48109 USA

**Keywords:** Hedgehog signaling, Enhancers, Machine learning, GLI

## Abstract

**Background:**

The Hedgehog (Hh) signaling pathway, acting through three homologous transcription factors (GLI1, GLI2, GLI3) in vertebrates, plays multiple roles in embryonic organ development and adult tissue homeostasis. At the level of the genome, GLI factors bind to specific motifs in enhancers, some of which are hundreds of kilobases removed from the gene promoter. These enhancers integrate the Hh signal in a context-specific manner to control the spatiotemporal pattern of target gene expression. Importantly, a number of genes that encode Hh pathway molecules are themselves targets of Hh signaling, allowing pathway regulation by an intricate balance of feed-back activation and inhibition. However, surprisingly few of the critical enhancer elements that control these pathway target genes have been identified despite the fact that such elements are central determinants of Hh signaling activity. Recently, ChIP studies have been carried out in multiple tissue contexts using mouse models carrying FLAG-tagged GLI proteins (GLI^FLAG^). Using these datasets, we tested whether a meta-analysis of GLI binding sites, coupled with a machine learning approach, could reveal genomic features that could be used to empirically identify Hh-regulated enhancers linked to loci of the Hh signaling pathway.

**Results:**

A meta-analysis of four existing GLI^FLAG^ datasets revealed a library of GLI binding motifs that was substantially more restricted than the potential sites predicted by previous in vitro binding studies. A machine learning method (kmer-SVM) was then applied to these datasets and enriched k-mers were identified that, when applied to the mouse genome, predicted as many as 37,000 potential Hh enhancers. For functional analysis, we selected nine regions which were annotated to putative Hh pathway molecules and found that seven exhibited GLI-dependent activity, indicating that they are directly regulated by Hh signaling (78 % success rate).

**Conclusions:**

The results suggest that Hh enhancer regions share common sequence features. The kmer-SVM machine learning approach identifies those features and can successfully predict functional Hh regulatory regions in genomic DNA surrounding Hh pathway molecules and likely, other Hh targets. Additionally, the library of enriched GLI binding motifs that we have identified may allow improved identification of functional GLI binding sites.

**Electronic supplementary material:**

The online version of this article (doi:10.1186/s12861-016-0106-0) contains supplementary material, which is available to authorized users.

## Background

The Hedgehog (Hh) signaling pathway is critical for embryonic organ development and adult tissue homeostasis across animal phyla [1–4]. In multiple tissue-specific settings, Hh signaling directs specific cell fate choices, controls tissue patterning and governs cell proliferation. In mammals, Hh signaling originates with any of three ligands (Sonic hedgehog (*Shh*), Indian hedgehog (*Ihh*) or Desert hedgehog (*Dhh*)) (for Review see [5]). Release of the lipid modified HH ligand has been shown to be facilitated by SCUBE in conjunction with the transmembrane protein Dispatched [6–9]. Once discharged, HH ligands interact with the Patched (PTCH1 or PTCH2) receptor protein and with Hh-binding proteins BOC, CDO and GAS1 on target cells to relieve PTCH-dependent inhibition of the Smoothened (SMO) transmembrane protein [10, 11]. HH ligands can also be sequestered by the Hedgehog-interacting protein (HHIP), which dampens signaling [12].

Hh-mediated signal transduction culminates in the nucleus, with the binding of zinc-finger transcription factors (GLI1, GLI2, GLI3) to target gene sequences [13, 14]. However, proteolytic processing determines whether the GLI proteins act as repressors or activators. GLI1, which is not processed, functions exclusively as a transcriptional activator and may act to amplify Hh signals [15]. GLI2 and GLI3 can be converted to a repressor form in the absence of Hh ligand. In the presence of the Hh ligand, this processing is inhibited, allowing full-length GLI proteins to traffic to the nucleus and activate gene expression [15–17]. Processing of GLI proteins requires passage through the cilia [13, 18]; the kinesin KIF7 helps to properly construct the cilium and is enriched at the cilium tip, along with GLI and SUFU (Suppressor of Fused) [19].

The Hh signaling pathway is regulated by both positive and negative feedback. Indeed, a number of Hh pathway components, including *Boc*, *Cdo*, *Gas1*, *Gli1*, *Hhip*, *Ptch1* and *Ptch2* are thought to be direct transcriptional targets of Hh signaling in multiple tissue contexts [12, 15, 20–29]. Thus, an important aspect of Hh pathway self-regulation is integrated at the level of the enhancers that control response of the pathway target genes to local Hh signaling levels. However, despite the high functional conservation of this pathway, surprisingly little is known about the enhancer elements that control self-regulation in any organism.

One way to identify Hh target enhancers is to perform chromatin immunoprecipitation (ChIP). Genetically modified mouse models carrying inducible FLAG-tagged GLI proteins have allowed analysis of GLI binding sites in vivo in several different tissue contexts. Four in vivo GLI binding studies, including three ChIP-chip analyses [26, 27, 29] and one ChIP-seq study [25], have been carried out using these models. Interestingly, examination of all four datasets for common GLI binding sites that are annotated to Hh pathway molecules reveals only three such sites (in *Gli1*, *Ptch1*, and *Ptch2* loci [15, 24, 28]) that are uniformly detectable. Several other established Hh pathway genes, including *Boc*, *Hhip*, *Gli2*, and *Hipk2*, appear to exhibit different GLI-bound genomic locations, depending on context, suggesting that each of these pathway components is regulated by multiple distinct genomic enhancers that have context-specific features.

Using ChIP studies on diverse tissues, it may be possible to eventually identify all of the multiple enhancers that control each target gene in every context. While a valuable goal, such analyses are currently expensive and time consuming and often technically challenging where the number of cells available for analysis is limiting, as in many developmental contexts. Importantly, computational methods can reveal sequence features that characterize enhancer activity. We therefore asked whether analysis of all existing GLI ChIP data could reveal common sequence features that might be used to empirically and globally predict functional enhancers *de novo*. A publicly available machine learning approach, kmer-SVM [30], was used to predict novel Hh enhancer regions. This tool uses a support vector machine (SVM) to determine sequence features (k-mer frequencies) that identify positive genomic regulatory regions [31]. SVMs are classifier algorithms that define a boundary between members of two different groups. Kmer-SVM calculates weights for sequence features that determine the effectiveness of that feature to distinguish between positive and negative regulatory regions. Once the features are determined, they can be used to identify novel enhancer regions not present in the original positive set. The strength of this approach is that it relies exclusively on short regions of DNA sequence (length 3–10 bp) which are in the size range of transcription factor binding sites (TFBS). Additionally, the organization of the k-mers within a sequence does not impact the score; this feature is consistent with the variable arrangement of TFBS in enhancers [32, 33].

Using the kmer-SVM tool [30], analysis of the four existing GLI binding datasets identified a set of k-mers that appeared to successfully predict potential GLI-regulated enhancers. Application of this set of k-mers to the mouse genome pinpointed over 37,000 potential enhancers. Several putative enhancers that were annotated to Hh pathway components were then tested for their ability to drive Hh-dependent activity in transfected cells. The functional significance of the GLI binding motifs (GBM) was also tested within each active enhancer by mutation. Of the nine predicted regulatory regions tested, seven (78 %) drove reporter expression in a GLI-dependent fashion. These findings substantially increase the number of functionally verified Hh enhancers found in Hh pathway molecules and validate the use of machine learning on ChIP data as a valuable tool to empirically predict likely Hh-dependent regulatory regions.

## Results and discussion

### Analysis of GLI^FLAG^ datasets to identify likely in vivo GLI transcription factor binding motifs

A previous in vitro analysis of GLI transcription factor binding resulted in the identification of a set of likely binding sites for this factor [34]. However, this spectrum of sites may not accurately represent GLI binding site preferences in vivo. To begin to examine this, we performed a meta-analysis of four existing GLI-ChIP datasets. All of these datasets utilize transgenic mice carrying FLAG tagged GLI1 (GLI1^FLAG^) or GLI3 (GLI3^FLAG^) in the ROSA26 locus, activated by Cre recombination, in four different tissue contexts: limb bud development (LD) [27], cerebellum development (CD), medulloblastoma (MB) resulting from Hh signaling overexpression [29], and neural progenitor cells (NP) [25]. An additional study of neural progenitors [26] was excluded from analysis since it contained a low number of significant peaks and mirrors the same experimental conditions as the NP dataset [25]. For each of the datasets, the reported percentage of ChIP peak sequences with GLI binding motifs (GBM) was as follows: LD 55 %, CD 26 %, MB 46 %, and NP 91 %. However, the definition of GBM was not the same across all datasets: one study allowed only two mismatches from the consensus [27] and others generated a GLI motif *de novo* based on the sequences of recovered peaks [25, 29].

**Fig. 1:**
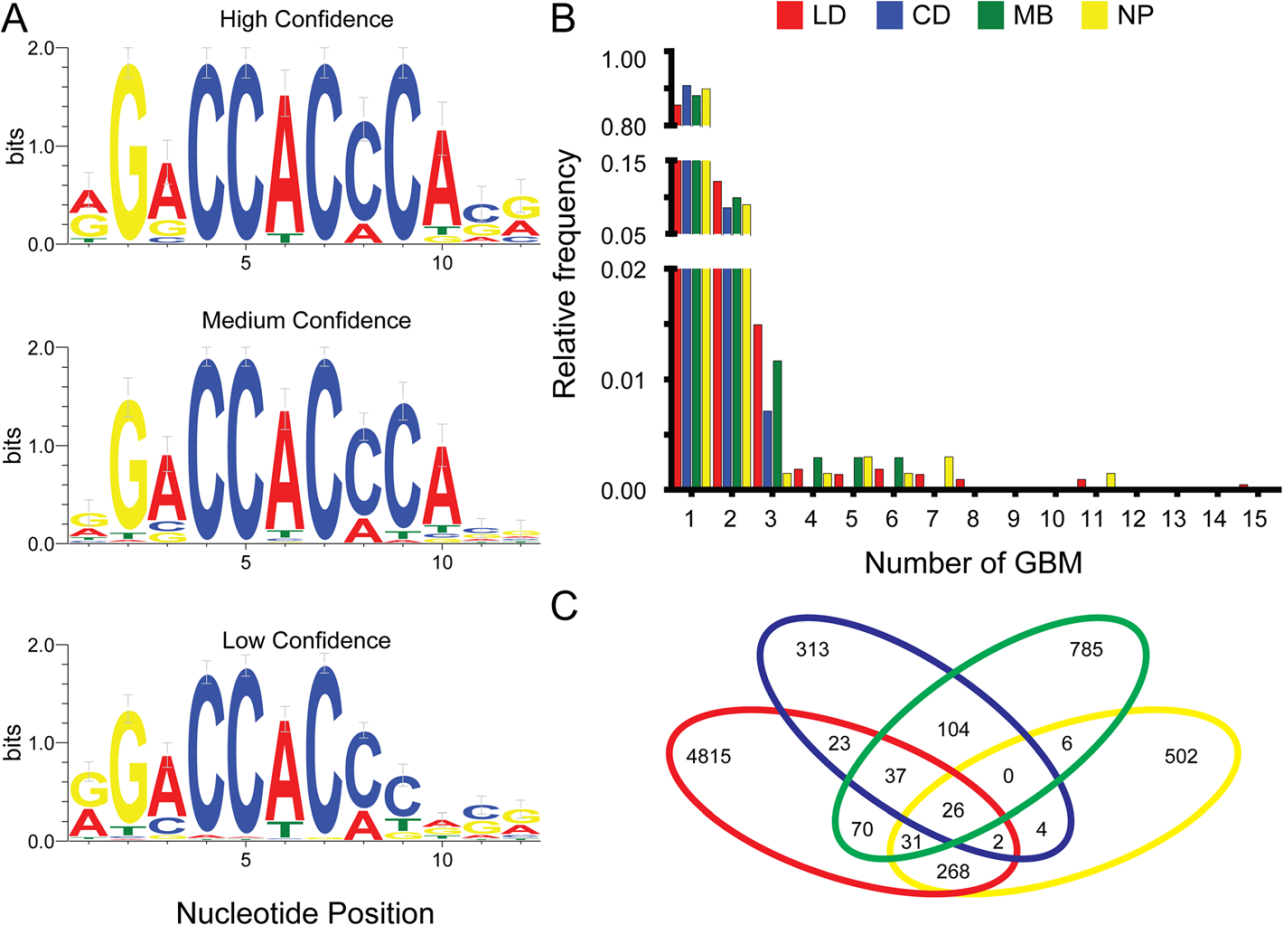
Definition of GLI binding motifs (GBM) and characterization of GLI^FLAG^ datastets. **a** Sequence logos (Weblogo) of 12-mer GBM. High confidence (HC), medium confidence (MC), and low confidence (LC) GBM are found in all four (HC), three or two (MC) or one (LC) datasets. **b** Relative frequency of peaks containing one or more GBM in the GLI^FLAG^ ChIP-chip LD (*red*), CD (*blue*), MB (*green*), and GLI^FLAG^ ChIP-seq NP (*yellow*) datasets. A high proportion of sequences contain only one GBM. **c** Overlap of sequences identified by all four GLI^FLAG^ datasets; only 26 individual peaks are found in all contexts

To collate the spectrum of GBM observed in all four datasets, we applied a *de novo* motif enrichment analysis to each dataset individually [35]. Sequences that contained at least one site that matched the *de novo* motifs were removed from the dataset. The remaining sequences were analyzed for residual motifs that resembled a GBM using DREME [36] and Tomtom [37] (see Methods). This resulted in 548 putative GBM (12-mers) (Additional file 1: Table S1), encompassing the range of GBM that are present in existing ChIP data. This set therefore represents a collection of likely genomic GLI binding sites, although some functional GLI binding sites in vivo could be absent from this set and some false positive sites may be included. Each 12-mer was classified as high confidence (HC), medium confidence (MC), or low confidence (LC) if it was found within sequences from all four datasets, two to three datasets, or one dataset, respectively. The sequence logos [38] for each classification, provided in Fig. 1a, show a nearly absolute representation of CCxC in positions 4–7 for all sites. Indeed, concordant (C and C or G and G) nucleotides at the 5^th^ and 7^th^ position were previously found to be required for GLI binding [39]. Interestingly, for high confidence sites, there is no variation at 5 of the 12 positions, including the 5^th^ and 7^th^ positions (xGxCCxCxCxxx).

Using the recommended matrix similarity score cutoff of 81 % overall matrix similarity to the optimal consensus GLI site as defined by the in vitro DNA binding assay [34] results in 1,432,161 putative GLI TFBS across the mouse genome. This is substantially more than the 191,745 found using the new GLI library defined by the in vivo ChIP studies. However, several of the sites predicted by the in vitro binding studies do not contain the concordant (C and C or G and G) nucleotides at the 5^th^ and 7^th^ position. Thus, the newly generated GLI library (Fig. 1a) may more accurately represent functional GBM.

According to this new library of 548 GBM, 41 % of LD, 27 % of CD, 32 % of MB, and 80 % of NP peaks contain putative GLI binding sites. For those sequences that contain a GBM, the vast majority contain only a single site (85.5 % LD, 90.7 % CD, 88.0 % MB, 89.8 % NP) (Fig. 1b). The overlap of genomic binding regions among datasets is shown in Fig. 1c; only 26 genomic coordinates are shared among all datasets (Additional file 2: Table S2). Since pathway components must respond to the Hh signal in all tissues, it might be expected that this common response would be integrated by a single enhancer. However, only three of the 26 shared regions are annotated to known Hh pathway components (*Gli1*, *Ptch1*, *Ptch2*). Indeed, for *Boc* and *Hhip*, distinct genomic GLI binding regions are found in different datasets. This result suggests that some of these genes may have multiple enhancers that work to transduce the Hh signal in different tissue contexts.

Given this apparent complexity in regulatory regions, we next asked whether the existing datasets of ChIP peaks might contain additional sequence information that could be used to predict the location of other Hh-responsive enhancers in the mouse genome. A machine learning approach was employed to test this question.

### Assessment of kmer-SVM performance and prediction

#### Kmer-SVM assessment of classification using GLI^FLAG^ datasets

For each GLI^FLAG^ dataset, only sequences with at least one GBM (wGBM, meaning *with GBM*) were used. This was done since a high proportion of the ChIP-chip datasets did not contain a putative GLI binding site as defined in the original papers (LD 55 %, CD 26 %, MB 46 %, and NP 91 %) or by our assessment (41 % of LD, 27 % of CD, 32 % of MB, and 80 % of NP peaks). Each individual dataset was submitted to kmer-SVM and the ability of each classifier to correctly label a candidate sequence as positive was assessed. Background sequences were randomly selected from the genome, but matched for GC content with the positive set.

Kmer-SVM randomly divides the data as follows: 80 % of the sequences are used as a training set and 20 % are used as a testing set. The ability of the classifier built with the training set to accurately identify the members of the remaining 20 % testing set is then assessed. This is repeated five times, each with a different random division of the data. Receiver operating characteristic (ROC) curves and precision recall curves (PRC) are used to assess the success of the classifier to correctly label regions in the testing set as positive (see Methods).

**Fig. 2:**
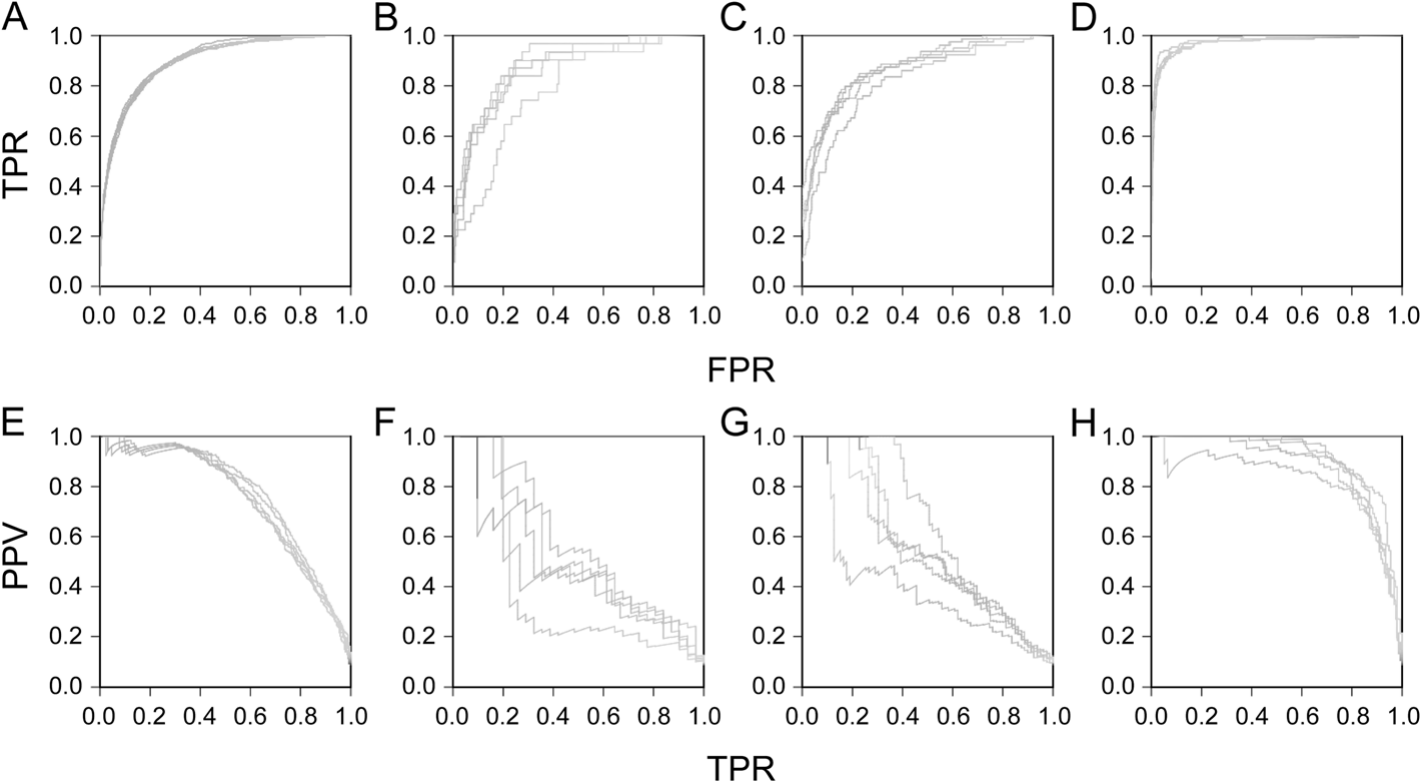
Assessment of classification capability of kmer-SVM trained GLI^FLAG^ datasets containing sequences with at least one GBM. For all curves, each dataset is randomly split into 80 % for training and 20 % for prediction and the prediction is repeated five times (*represented by individual lines*). Plots assess the likelihood that the specified classifier can successfully predict sequences that have at least one GBM as positive or negative. **a**-**d** ROC plots depicting true positive rates (TPR) and false positive rates (FPR). Area under the curve (AUC) scores as calculated by kmer-SVM are: 0.89 for LDwGBM (**a**), 0.85 for CDwGBM (**b**), 0.86 for MBwGBM (**c**) and 0.97 for NPwGBM (**d**) datasets. **e**-**h** Precision recall curves depicting the positive predictive value (PPV), calculated as true positive / (true positive + false positive), versus the TPR. AUC of 0.75 for LDwGBM (**e**) and 0.88 for NPwGBM (**h**) indicate reasonable confidence in the classification while AUC of 0.49 for CDwGBM (**f**) and 0.55 for MBwGBM (**g**) indicate a low probability that the region is correctly labeled when the sequence is classified as positive

ROC curves display the cumulative distribution of the true positive rate compared to the false positive rate. This characteristic assesses how well the classifier is able to label the positive sequences from the test set. The area under the curve was 0.898 for LDwGBM (Fig. 2a), 0.856 for CDwGBM (Fig. 2b), 0.862 for MBwGBM (Fig. 2c) and 0.976 for NPwGBM (Fig. 2d). Thus, the classifier performs best in LD and NP datasets.

PRC displays the predictive value against the true positive rate and represents the accuracy of the labeling. The PRC plots indicated high values for LDwGBM (AUC = 0.753) and NPwGBM (AUC = 0.880) but low values for CDwGBM (AUC = 0.490) (Fig. 2f) and MBwGBM (AUC = 0.546) (Fig. 2e-h). The ROC and PRC plots for LDwGBM and NPwGBM datasets suggested that the classifier sequence features used were able to distinguish between positive and negative groups with a low level of false labeling.

#### Predictions

The classifiers for LDwGBM and NPwGBM were then individually run on 600 bp of sequence centered on every GBM in the mouse genome (191,745, as determined using the new GBM from in vivo data, described above). Use of both the LDwGBM and NPwGBM datasets for prediction incorporated data from the GLI1^FLAG^ (predominately activator) and GLI3^FLAG^ (predominantly repressor) transcription factors in two diverse contexts (neuronal precursor and limb development).

The length of 600 bp was selected based on motif enrichment analysis of the LD and NP datasets using MEME-ChIP [40] and Centrimo [41]. This analysis showed that, within the ChIP-chip LD dataset, enrichment for the location of GLI motifs (green line) has a broad profile that spans 200 bp to either side of the midpoint (Additional file 3: Figure S1A). The GLI motif has a narrower profile in the NP data, a feature that is expected for ChIP-seq (Additional file 3: Figure S1B). The profile for the Sox motif (blue line), an established tissue specific GLI cofactor [25], shows an enrichment peak that is centered around 200 bp on either side of the midpoint (Additional file 3: Figure S1B) and suggests that cofactors for Hh may reside outside of the immediate vicinity of a GLI binding site. We therefore used 600 bp to capture both common Hh features as well as potential context specific sequence.

**Fig. 3:**
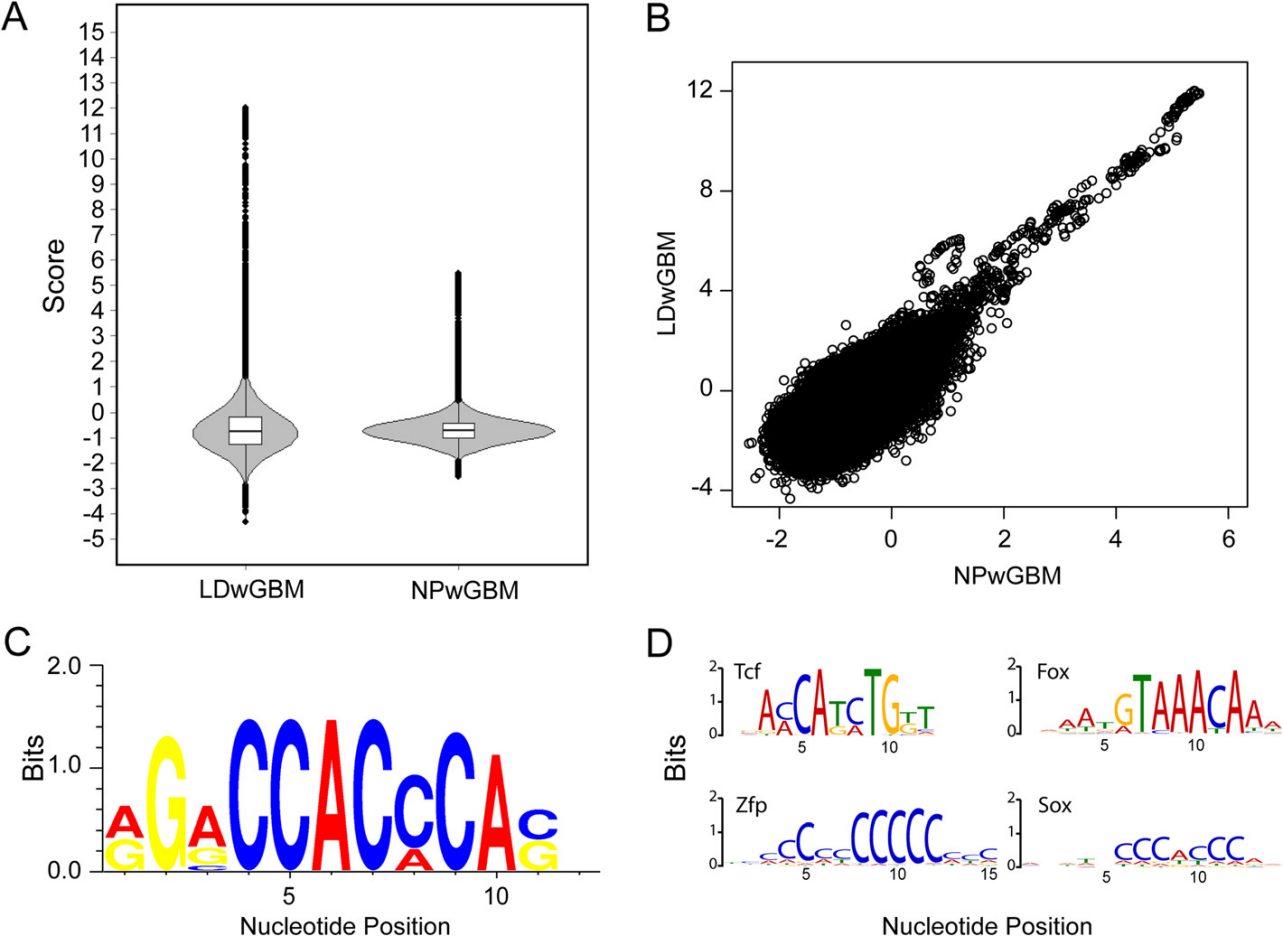
Assessment of genomic kmer-SVM predictions using classifiers trained on LDwGBM and NPwGBM datasets. **a** All genomic sequences matching the restricted 548 GBM 12-mers (wGMB) were identified and the 600 bp surrounding each GBM were assessed and scored using the kmer-SVM classifier that was trained on each of the two datasets. **b** Correlation plot depicting the relationship between LDwGBM and NPwGBM scores; scores >1 are highly correlated in the two datasets. **c** GLI motif generated from overlapping high weighted k-mers shared between LDwGBM and NPwGBM classifiers. **d** High weighted k-mers (identified by Tomtom) represented in either LDwGBM (Tcf and Zfp) or NPwGBM (Fox and Sox)

For the LDwGBM classifier, scores ranged from −4.33 to 12.00 with 18.4 % of the 191,745 analyzed genomic regions scoring as positive (Score > 0). The NPwGBM results ranged from −2.54 to 5.48 with 5.7 % positive (Fig. 3a; Additional file 4: Table S3). The categorization of a sequence is dependent on the sign of the score and the weight of the value is less important than the ranking. Overall, the correlation between scores for individual genomic regions calculated by the LDwGBM and NPwGBM classifiers is poor (0.68 Pearson) (Fig. 3b). However, if only sequences with positive scores are considered, the correlation improves (0.85 Pearson). If scores are restricted to values indicating only the high confidence scores (calculated posterior probabilities = 1.0, Additional file 5: Figure S2), the values are very well correlated (0.96 Pearson). In total, 8627 genomic regions were predicted as Hh enhancers by both classifiers. Of those, 1198 regions (14 %) overlapped at least one peak in the four GLI^FLAG^ datasets. Among high confidence scores (LDwGBM: 5951 ≥ 1, NPwGM: 547 ≥ 1) 528 genomic regions were shared between the two datasets and 187 of these (35 %) overlapped with peaks from at least one of the four GLI^FLAG^ datasets. All of the scored regions are listed in Additional file 4: Table S3.

#### Evaluation of predictions

To assess whether kmer-SVM predictions were likely to represent Hh enhancers, we examined whether the predicted genomic regions overlapped publically available open chromatin and enhancer histone marks in tissues that were likely to be transducing Hh signals. We first examined the DNaseI hypersensitive profile collected from mouse mesoderm at E11.5 at genomic regions that were predicted with high confidence as positive (1 ≤ score; posterior probability = 1) or negative (−1 ≥ score; posterior probability = 0) (Additional file 5: Figure S2). A significantly higher proportion of overlap was found with the predicted positive regions than with predicted negative regions (Z-Score = 2.8332; *p*-value < 0.05) (Additional file 6: Table S4). We also examined publically available H3K4me1, H3K4me3, and H3K27ac ENCODE data collected from heart and liver at E14.5. Monomethylated H3K4 (H3K4me1) and histone H3 acetyl Lys27 H3K27ac [42] were used as enhancer markers while trimethylated H3K4 (H3K4me3) was expected to be depleted in enhancer regions [43]. Although Hh signaling is active during early development of both tissues, available in situ analysis for GLI1 (GenePaint: EN1215) [44, 45] shows GLI1 expression in liver but not heart at E14.5 (Additional file 7: Figure S3). Thus, we expected signals for both H3K4me1 (poised enhancer) and H3K27ac (active enhancer) to be enriched in the predicted positive regions in liver but not heart at this time point. Indeed this was the case: H3K4me1 (Z-Score = 2.5511; *p*-value < 0.01) and H3K27ac (Z-Score = 8.076; *p*-value <0.01), with no significant difference in H3K4me3 when predicted positive regions were compared to predicted negative regions. As expected, the heart data did not show enrichment for H3K4me1 or H3K27ac. Together, the results (summarized in Additional file 6: Table S4) are consistent with the conclusion that the kmer-SVM classification correctly identifies Hh enhancer regions.

Next, we evaluated the sequence features, or k-mers, that kmer-SVM identified as primary components of Hh enhancer regions. The weights of k-mers are calculated during the SVM training and reflect the contribution of the k-mer to categorization of a sequence. Weights can be positive or negative and the sum of the weights of iterative k-mers across a sequence comprise the overall score of that sequence. Not surprisingly, alignment of k-mers with high scoring weights shared between both datasets returned a motif that strongly resembles the GBM (Fig. 3c). Unique high weighted k-mers that occurred in each individual dataset represented potential context specific features. An E-box motif was identified for the LDwGBM dataset while a Sox motif was returned for NPwGBM (Fig. 3d). Negative weights that occurred in both datasets include AC and ACC repeats as well as other C rich sequences.

### Functional verification of GLI-dependent enhancer activity

Predicted genomic regions were annotated to the two nearest genes using GREAT [46]. Because our goal was to identify enhancers for Hh pathway components, we selected a subset of predictions that were positive in both the LDwGBM and NPwGBM datasets and that were annotated to members of the GO:0007224 Smoothened signaling pathway gene set. Because Hh pathway components are required for active Hh signaling, we reasoned that enhancers annotated to these genes would be more likely to function in any tissue that transduces Hh signal. Therefore, high scoring regions annotated to different members of the GO:0007224 gene set that were readily cloned were functionally tested for enhancer activity. Two previously known Hh enhancers for *Ptch1* and *Ptch2* appeared on this list [24, 28]. Interestingly, an established *Gli1* regulatory region was not predicted [15]. The test set consisted of genomic regions annotated to *Boc*, *Gli3*, *Hhip*, *Hipk2*, *Ptch1*, *Scube1*, *Shh*, and *Tgfbr2*. An additional region, annotated to *Dpp6* (near *Shh*) was also tested (Table 1).

**Table 1:** Assessment of predicted Hh enhancer regions

Annotated gene	Genomic coordinates (mm9)	Hh responsive	LD	CD	MB	NP
*Ptch2*	chr4:116,767,757-116,769,455	+	+	+	+	+
*Boc*	chr16:44,502,136-44,503,346	+	-	+	+	-
*Dpp6*	chr5:27,248,056-27,249,266	+	+	-	-	-
*Gli3*	chr13:15,764,694-15,765,904	-	-	-	-	-
*Hhip*	chr8:82,838,195-82,839,405	+	+	-	-	+
*Hipk2*	chr6:38,614,001-38,615,211	+	-	-	-	-
*Ptch1*	chr13:63669992-63671202	+	+	-	+	+
*Scube1*	chr15:83503053-83504263	+	-	-	-	-
*Shh*	chr5:28832033-28833243	-	-	-	-	-
*Tgfbr2*	chr9:116,151,184-116,152,394	+	-	-	-	-

Seven of the nine regions predicted to be GLI-driven enhancers were indeed determined to be Hh responsive and GLI binding site dependent in a cell culture assay. Overlap of the predicted regions with peaks from the GLI^FLAG^ ChIP datasets (LD, CD, MB, NP) is indicated by the plus sign. *Boc*, *Hipk2*, *Scube1* and *Tgfbr2* were predicted by kmer-SVM and found to be positive, even though those regions do not overlap any of the peaks identified in the LD or NP datasets used to generate the kmer-SVM classifier

The nine genomic regions were screened for Hh responsiveness in a cell culture assay that has been previously used to validate Hh enhancers [47, 48]. In this assay, C2C12 cells are transiently transfected with constructs containing the putative Hh regulatory region cloned upstream of a minimal promoter that drives luciferase expression (see Methods). To induce a Hh response, enhancer reporter constructs were co-transfected with a plasmid that drives constitutive expression of GLI1 in mammalian cells [26]. For those candidate enhancers that demonstrated apparent Hh activation, GLI-dependent activity was confirmed by retesting after mutagenesis of the GLI binding sites (GKO). Either complete loss of enhancer activity or attenuation of response in GKO sequence was considered GLI-dependent. The established Hh enhancer region for *Ptch2* was used as a positive control [28].

**Fig. 4:**
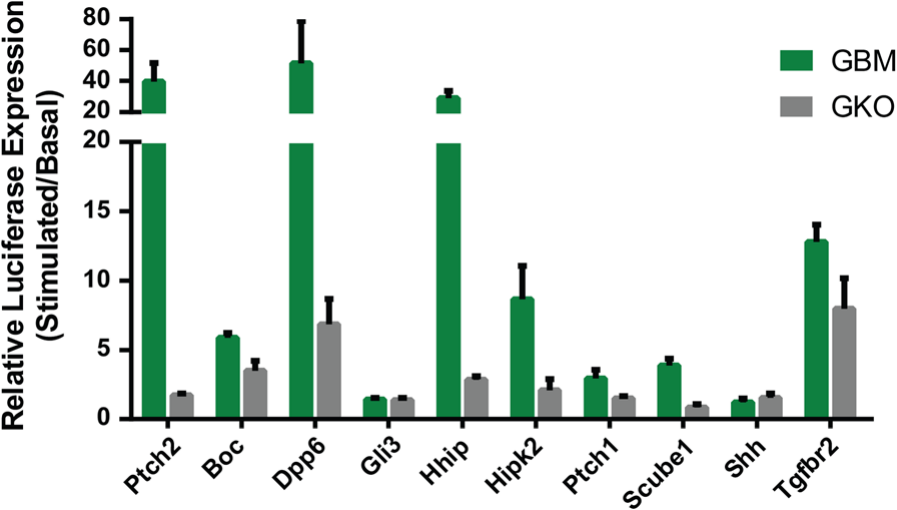
Functional verification of GLI-dependent enhancer activity. Putative regulatory regions were cloned upstream of a minimal promoter co-transfected into C2C12 cells, along with a GLI1 expression vector. Relative activity is plotted (stimulated/basal). The Ptch2 region is used as a positive control. Novel regions annotated to the *Boc*, *Dpp6*, *Hhip*, *Hipk2*, *Ptch1*, *Scube1*, and *Tgfbr2* loci exhibit upregulation in response to GLI1 co-transfection (*green*). Dependence on GLI was assessed by mutating all putative GLI TFBS (GKO) within the sequence and retesting in the assay (*gray*)

For the 9 regulatory regions annotated to Hh pathway component genes, 7 exhibited Hh activation that was directly dependent on a GLI binding site (Fig. 4). The *Ptch2* positive control region showed a complete loss of Hh response after mutation of the GLI site (Fig. 4) as did the regulatory regions annotated to *Hhip*, *Hipk2*, *Ptch1*, and *Scube1*. Regions annotated to *Boc*, *Dpp6*, and *Tgfbr2* showed a significant decrease in Hh activation upon GLI TFBS mutation, but not a complete loss of response. This suggests that additional regulatory inputs influence the activity of these enhancers. Neither the *Gli3* nor *Shh* region exhibited Hh dependent enhancer activity. However, we cannot rule out the possibility that these regions might be positive if examined in a different cellular context [26].

Two previous *in silico* methods have been described for the identification of Hh-regulated enhancers in vertebrates: Enhancer Element Locator (EEL) and Module Cluster Analysis (MCA). EEL analyzes the estimated energy of a single transcription factor binding event, as well as the possible interaction between adjacent, highly conserved transcription factor binding sites, to detect potential enhancers [34]. In contrast, MCA utilizes Poisson modeling to determine the relative enrichment of binding sites in highly conserved, non-coding sequence and, thereby, identify putative enhancers [26]. While both of these methods have had some success (~25 %) in detecting putative Hh-regulated enhancers, a disadvantage to these approaches is that the search is limited to regions of high sequence conservation and to regions close to promoters. In contrast, kmer-SVM approach used here employs a genome-wide empirical analysis to locate regions that contain sequence features predictive of Hh enhancer function. Though these predictions miss one of the three known Hh pathway enhancers [15] indicating that the algorithm does not capture all Hh-driven enhancers, the high success rate (78 %) of the kmer-SVM predictions far exceed the previous prediction rates for EEL or MCA. It is important to note, however, that the predictions tested here are all for pathway genes, which may have a unique signature. It would be necessary to test additional enhancers in tissue-specific assay systems (e.g., transgenic mice) to determine the overall success of this method in identification of tissue-specific enhancers.

**Fig. 5:**
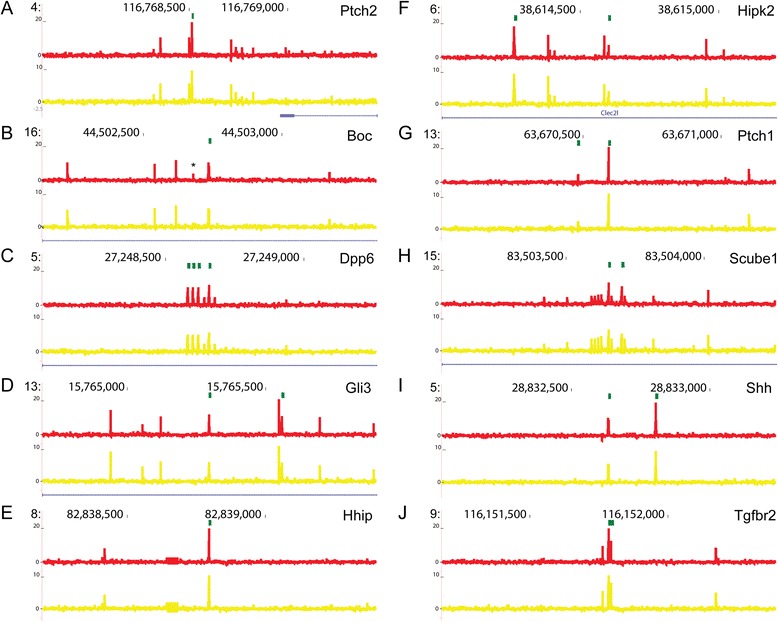
K-mer weights plotted across sequences that show enhancer activity. Diagrams were generated in UCSC Genome browser and show coordinate information for regions annotated to *Ptch2* (**a**), *Boc *(**b**), *Dpp6* (**c**), *Gli3* (**d**),* Hhip *(**e**), *Hipk2* (**f**),* Ptch1 *(**g**), *Scube1* (**h**), *Shh *(**i**) and *Tgfbr2* (**j**). Green boxes represent GBM. Weights for LDwGBM and NPwGBM are represented by the red and yellow lines, respectively. Refseq gene annotations are represented in blue. A putative Krox-20 TFBS (*) that has a high weight in the LDwGBM classifier but not the NPwGBM classifier occurs in the sequence annotated to *Boc*. Note that most sequences show weighted k-mers located several hundred bp from the central GBM, suggesting that sequence motifs that predict Hh enhancer activity may be distributed

To examine the impact of k-mers that contribute to predictions of the positive regions, weights were plotted across each of the sequences. The *Ptch2* sequence (Fig. 5a), a known enhancer region [28], contained matching profiles for 8-mers predicted from LDwGBM (red) and NPwGBM (yellow) that are GC rich and similar to the GBM k-mers However, mutation of the single GLI TFBS (Fig. 5b, green box) ablates the Hh response, indicating that the presence of this GBM is required to transduce Hh signaling. Mutation of the GBMs annotated by the green boxes for *Hhip*, *Hipk2*, *Ptch1*, and *Scube1* (Fig. 5e, f, g, h) is also sufficient to abrogate Hh signal transduction. For *Boc*, *Dpp6*, and *Tgfbr2*, which show enhancer activity that remains after ablation of the GBM (Fig. 5b, c, j), there were no sequence characteristics that were indicative of a shared feature responsible for this remaining response to induction by GLI1. The Boc profile was the only one that contained a high weighted k-mer (annotated with an asterisk) that was unique to the LDwGBM (red) profile. This k-mer was similar to a Krox motif (Tomtom *p*-value < 0.004) [37] and may be enriched in the LDwGBM dataset as a context specific transcription factor, since it has roles in limb development [49, 50]. In general, most of the tested regions contain distributed high weighted 8-mers in addition to the central GBM and had profiles that contained consistent peaks in both LDwGM and NPwGBM datasets (Fig. 5).

## Conclusions

We have utilized the kmer-SVM machine learning approach to examine four existing GLI ChIP databases and to generate classifiers that can empirically predict functional Hh enhancers from genomic DNA. The analysis was facilitated by a new GBS library generated from a meta-analysis of genomic regions identified in in vivo binding studies [25–27, 29]. When compared to the previous library derived from in vitro binding studies [34], this new GBS library resulted in the identification of a subset (approximately 10 %) of potential GLI transcription factor binding sites across the mouse genome. Of nine predicted Hh target regulatory regions tested, seven were functionally verified as GLI-dependent. All of the tested regions were annotated to genes believed to be components of the Hh pathway and important determinants of the Hh response. Given the high success rate of Hh enhancer prediction in this small scale screen (78 %), it is quite possible that a large number of the other 37,000 predicted regions (Score > 0 in Additional file 4: Table S3) may harbor Hh enhancer activity.

## Methods

### Computing resources

Except where otherwise indicated, all computational steps were performed using custom Perl and R scripts.

### Publically available datasets

Genomic analysis was conducted on chromosomes 1 to 19, X and Y of mouse build mm9. Mouse ENCODE data [51] comprising open chromatin DNaseI data that was collected at embryonic day 11.5 in the mesoderm and histone (H3K4me1, H3K4me3, and H3K27ac) data collected from embryonic day 14.5 for heart and liver were downloaded from the UCSC genome repository (goldenPath).

### Definition of putative GLI binding motifs

The library of putative GLI binding motifs (GBM) was compiled using *de novo* motif analysis [35] on each of the individual GLI^FLAG^ datasets iteratively. Sequences that contained a GBM were removed from the dataset and the remaining sequences were analyzed for enriched motifs using DREME [36]. If Tomtom [37] returned a GLI motif, the dataset was reanalyzed using HOMER [35]. The process continued until no residual GBM remained enriched in the dataset. Confidence in the GBM was classified as high (HC) if it was shared across sequences from all four GLI^FLAG^ datasets, medium (MC) if it was found in two or three datasets, and low (LC) if it only occurred in one.

### kmer-SVM parameters and evaluation of classifiers

Training was run on the Beer lab webserver (http://kmersvm.beerlab.org/), using a k-mer of length of 8.

Performance of the classifier built by kmer-SVM’s training algorithm was assessed using Receiver Operating Characteristic (ROC) and Precision-Recall curves (PRC) generated within the kmer-SVM program. True positive, true negative, false positive and false negative counts were generated by segregating the sets of positive and negative sequences into a training set (80 % of the sequences) and a testing set (the remaining 20 % of the sequences). Each member (individual sequence) of the testing set that is correctly annotated as positive increases the true positive count while an incorrect prediction of a positive sequence as negative increases the false negative count. ROC curves asses the sensitivity and specificity of the classifier output. A steep curve with a high area under the curve (AUC) indicates a high true positive rate and a low false positive rate. PRC evaluate the accuracy and relevance of the classifier output. A high AUC indicates that the results have a low false positive rate (high precision) and a low false negative rate. The trained SVM is evaluated by assessing its ability to classify the testing set correctly. The classifier was assessed five times by resetting members in the training set and testing set.

### Cloning of putative enhancer regions

Putative enhancers were amplified from C57BL/6 genomic DNA (supplied by Jackson Laboratory) using template-specific PCR primers (Additional file 8: Table S4). A CACC extension was added to the end of one primer to facilitate directional cloning. PCR fragments were cloned into the pENTR/D-TOPO vector using the standard kit (Invitrogen) and then shuttled into the pGL3-Promoter luciferase vector (Promega) using the Gateway® cloning system (Invitrogen). QuikChange mutagenesis (Stratagene) was used to mutate putative GLI binding sites by replacing the C in the 6^th^ position to a G.

### Luciferase assay

C2C12 cells (35,000) were plated per well on 12-well plates (10 % fetal bovine serum treated with penicillin, streptomycin and glutamate). After 24 h, cells were transfected, using lipofectamine, with 400 ng of the construct containing the putative enhancer region plus either a control vector or GLI1 (in equal molecular weight). *Renilla* (Promega pRL-CMV) was also included to normalize transfection efficiency. After an additional 24 h, cell media was changed to no serum to promote ciliogenesis [52]. Cell lysate was collected after 48 h and measured for luciferase activity using the Dual-Luciferase® Reporter Assay System (Promega) on a Perkin Elmer Wallac Victor3 1420 Multilabel Counter. Three experimental replicates were collected for each condition.

## Additional files

Additional file 1: Table S1. GLI binding motif 12-mers. Library of 12-mers enriched in GLI^FLAG^ datasets. Motifs were considered high confidence (HC) if the 12-mer occurred in all four GLI^FLAG^ datasets, medium confidence (MC) if it occurred in 2 or 3, or low confidence (LC) if it only occurred in one dataset. (XLSX 17 kb) Additional file 2: Table S2. Peak coordinates that overlap between all four GLI^FLAG^ datasets. The 26 regions that have shared peaks across all GLI^FLAG^ datasets. Peaks are annotated to nearest genes. (XLSX 9 kb) Additional file 3: Figure S1. Determination of sequence length buffer surrounding the GBM. Plots depicting the positional distribution of the best GLI motif (green) were generated by submitting 300 bp of sequence surrounding the center of each peak to Centrimo. (A) LDwGBM shows a broad profile for the best GBM, consistent with ChIP-chip data. (B) The profile for the ChIP-seq sequences from NPwGBM is more narrow and suggests that most of the GBM fall within 240 bp around the center of the peak. In neural precursor cells, the motif for the GLI cofactor, Sox, has a profile that contains a central apex plus two additional summits at a distance of 240 bp on either side of the peak. This suggests that context-specific TF binding may occur outside the central peak region. (TIF 15871 kb) Additional file 4: Table S3. GLI^FLAG^ dataset kmer-SVM scores. kmer-SVM scores for LDwGBM and NPwGBM datasets. (XLSX 20714 kb) Additional file 5: Figure S2. Posterior probability of kmer-SVM scores. Plots depicting the posterior probabilities assigned to scores for both (A) LDwGBM and (B) NPwGBM datasets. The graphs indicate that scores above 1 have a high confidence of being Hh regulatory regions. (TIF 14263 kb) Additional file 6: Table S4. Overlap of predicted high confidence positive and negative regions with embryonic open chromatin. Tabulation of the number of genomic regions predicted by both LDwGBM and NPwGBM that are classified with high confidence as Hh enhancer regions or as nonregulatory regions that overlap with mesoderm DNaseI (E 11.5) or enhancer markers (E14.5). (XLSX 9 kb) Additional file 7: Figure S3. Expression of GLI1 within E14.5 mouse embryo. In situ hybridization of GLI1 (image from genepaint.org, EN1215) showing active Hh signaling at E14.5 in liver but not heart. (JPG 216 kb) Additional file 8: Table S5. PCR primers for amplification of mouse genomic regions. Mouse genomic coordinates (mm9) for primer sequence used to amplify candidate regions. (XLSX 10 kb)

## Abbreviations

E14.5: embryonic day 14.5 (mouse)
GBM: GLI binding motif
Hh: Hedgehog
HPC: Hedgehog pathway component
SVM: support vector machine
TFBS: transcription factor binding sites
