# Supplementary figures and images for: Computational prediction and experimental validation of novel Hedgehog-responsive enhancers linked to genes of the Hedgehog pathway
Source: BMC Dev Biol. 2016 Feb 24;16:4. doi: 10.1186/s12861-016-0106-0 (PMC4765071; doi:10.1186/s12861-016-0106-0)

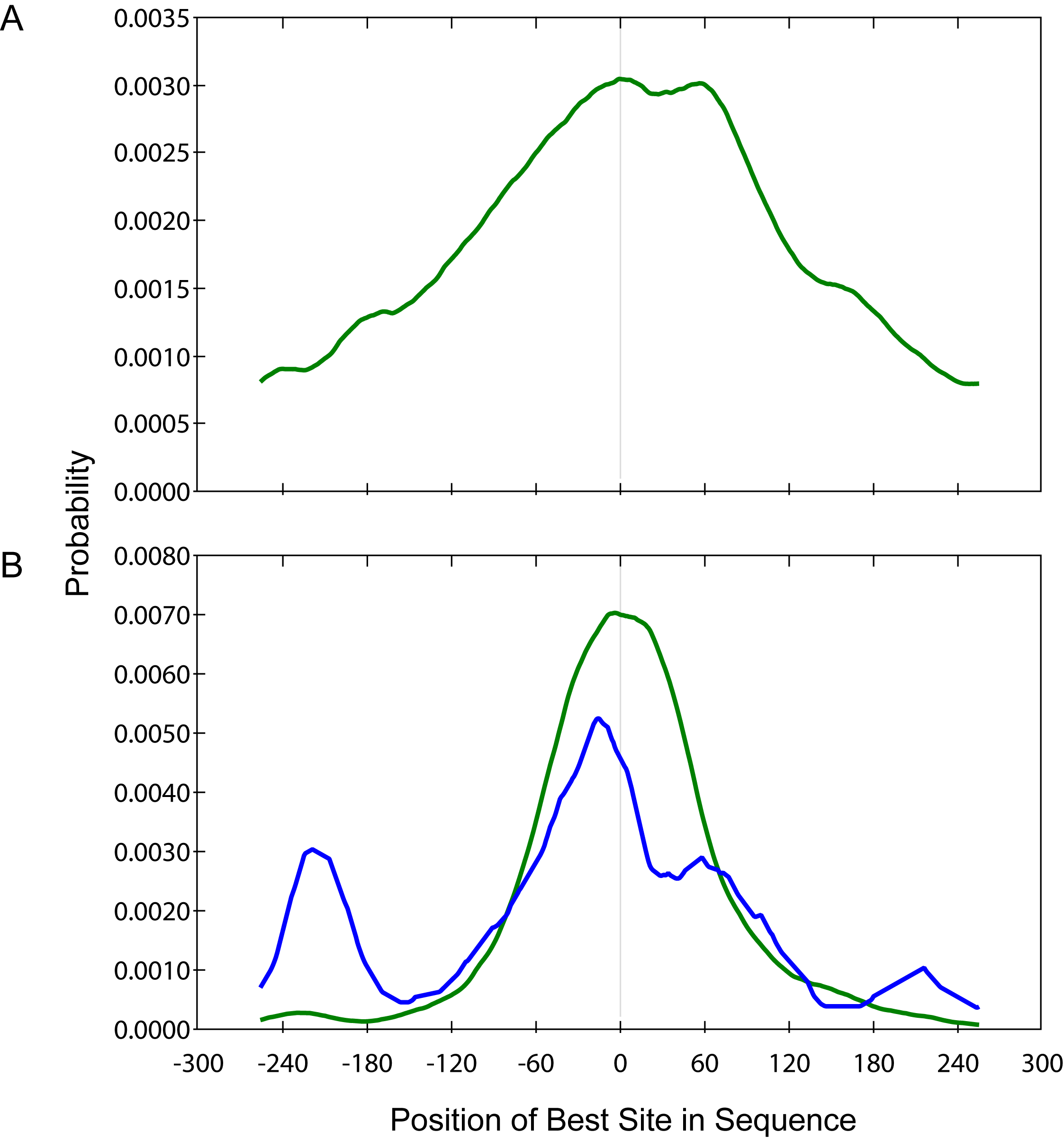

Supplement: Additional file 3: Figure S1. — Determination of sequence length buffer surrounding the GBM. Plots depicting the positional distribution of the best GLI motif (green) were generated by submitting 300 bp of sequence surrounding the center of each peak to Centrimo. (A) LDwGBM shows a broad profile for the best GBM, consistent with ChIP-chip data. (B) The profile for the ChIP-seq sequences from NPwGBM is more narrow and suggests that most of the GBM fall within 240 bp around the center of the peak. In neural precursor cells, the motif for the GLI cofactor, Sox, has a profile that contains a central apex plus two additional summits at a distance of 240 bp on either side of the peak. This suggests that context-specific TF binding may occur outside the central peak region. (TIF 15871 kb) [file 12861_2016_106_MOESM3_ESM.tif]

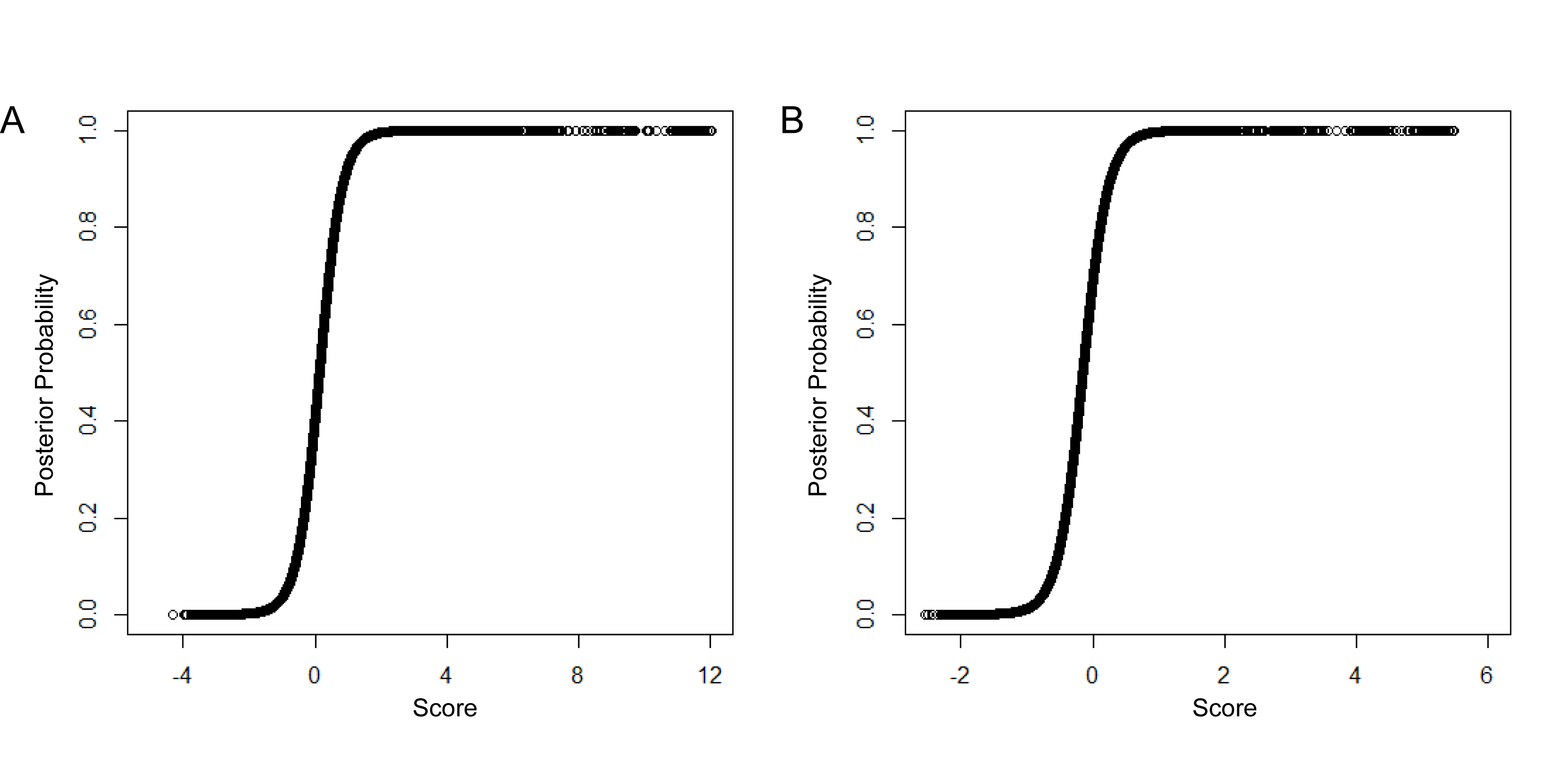

Supplement: Additional file 5: Figure S2. — Posterior probability of kmer-SVM scores. Plots depicting the posterior probabilities assigned to scores for both (A) LDwGBM and (B) NPwGBM datasets. The graphs indicate that scores above 1 have a high confidence of being Hh regulatory regions. (TIF 14263 kb) [file 12861_2016_106_MOESM5_ESM.tif]

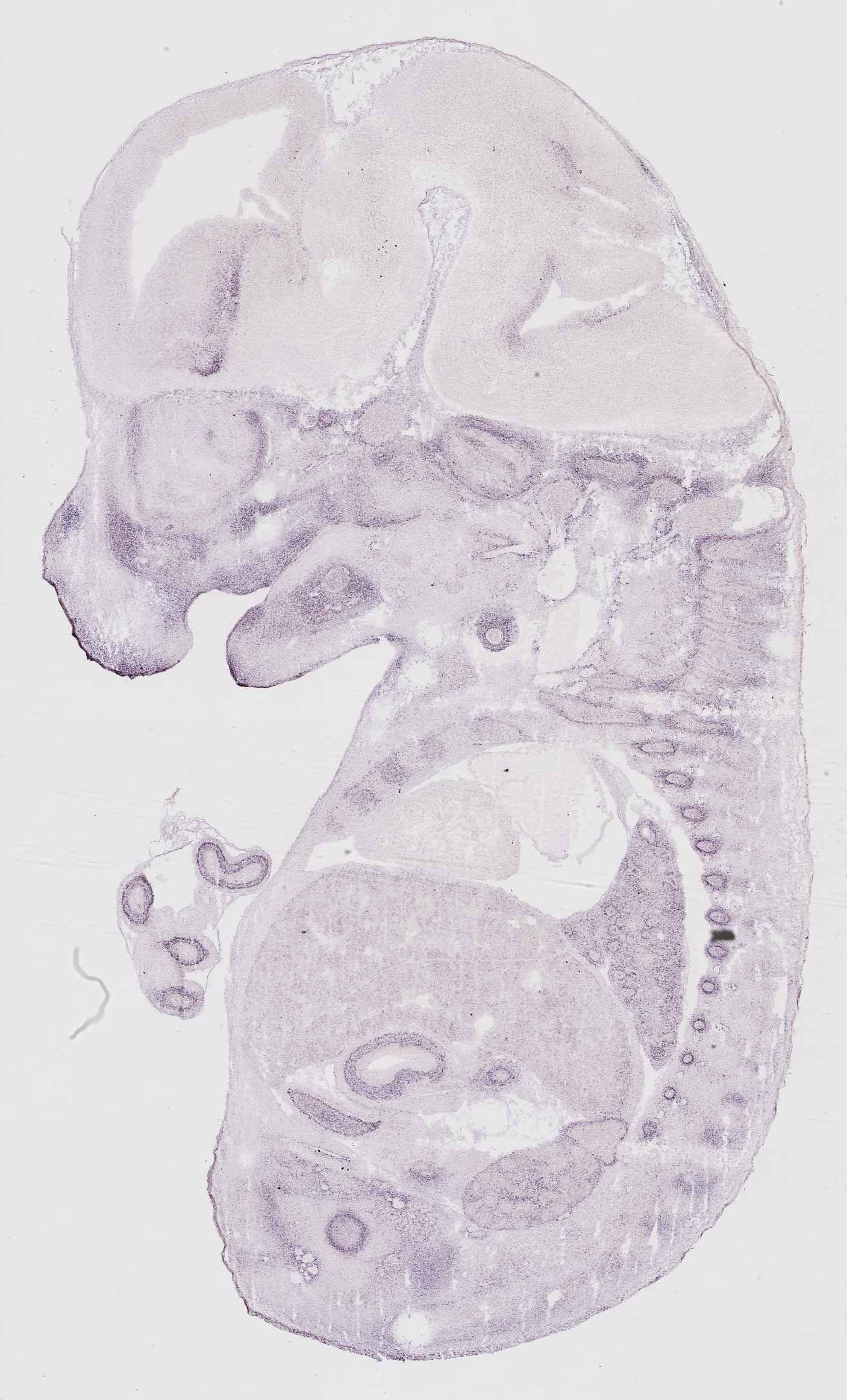

Supplement: Additional file 7: Figure S3. — Expression of GLI1 within E14.5 mouse embryo. In situ hybridization of GLI1 (image from genepaint.org, EN1215) showing active Hh signaling at E14.5 in liver but not heart. (JPG 216 kb) [file 12861_2016_106_MOESM7_ESM.jpg]
